# Modulation of brain activation during executive functioning in autism with citalopram

**DOI:** 10.1038/s41398-019-0641-0

**Published:** 2019-11-11

**Authors:** Robert H. Wichers, James L. Findon, Auke Jelsma, Vincent Giampietro, Vladimira Stoencheva, Dene M. Robertson, Clodagh M. Murphy, Grainne McAlonan, Christine Ecker, Katya Rubia, Declan G. M. Murphy, Eileen M. Daly

**Affiliations:** 1 Department of Forensic and Neurodevelopmental Sciences, The Sackler Centre for Translational Neurodevelopment, Institute of Psychiatry, Psychology and Neuroscience, King’s College London, London, UK; 2 Behavioural and Developmental Psychiatry Clinical Academic Group, South London and Maudsley NHS Trust, London, UK; 3 VU University Medical Center, Amsterdam, The Netherlands; 4 Department of Neuroimaging, Institute of Psychiatry, Psychology and Neuroscience, King’s College London, London, UK; 5 Department of Child and Adolescent Psychiatry, Psychosomatics and Psychotherapy, University Hospital Frankfurt am Main, Goethe-University Frankfurt am Main, Frankfurt, Germany; 6 Department of Child & Adolescent Psychiatry, Institute of Psychiatry, Psychology and Neuroscience, King’s College London, London, UK

**Keywords:** Molecular neuroscience, Predictive markers

## Abstract

Adults with autism spectrum disorder (ASD) are frequently prescribed selective serotonin reuptake inhibitors (SSRIs). However, there is limited evidence to support this practice. Therefore, it is crucial to understand the impact of SSRIs on brain function abnormalities in ASD. It has been suggested that some core symptoms in ASD are underpinned by deficits in executive functioning (EF). Hence, we investigated the role of the SSRI citalopram on EF networks in 19 right-handed adult males with ASD and 19 controls who did not differ in gender, age, IQ or handedness. We performed pharmacological functional magnetic resonance imaging to compare brain activity during two EF tasks (of response inhibition and sustained attention) after an acute dose of 20 mg citalopram or placebo using a randomised, double-blind, crossover design. Under placebo condition, individuals with ASD had abnormal brain activation in response inhibition regions, including inferior frontal, precentral and postcentral cortices and cerebellum. During sustained attention, individuals with ASD had abnormal brain activation in middle temporal cortex and (pre)cuneus. After citalopram administration, abnormal brain activation in inferior frontal cortex was ‘normalised’ and most of the other brain functional differences were ‘abolished’. Also, within ASD, the degree of responsivity in inferior frontal and postcentral cortices to SSRI challenge was related to plasma serotonin levels. These findings suggest that citalopram can ‘normalise’ atypical brain activation during EF in ASD. Future trials should investigate whether this shift in the biology of ASD is maintained after prolonged citalopram treatment, and if peripheral measures of serotonin predict treatment response.

## Introduction

Autism spectrum disorder (ASD) is a neurodevelopmental condition that is characterised by social communication deficits and repetitive or stereotypical behaviours^1^. It has been estimated that 29% of individuals with ASD in the UK are prescribed psychotropic medications^2^. Antidepressants, mainly selective serotonin reuptake inhibitors (SSRIs), are prescribed in 6% of people with ASD in the UK^2^, and are the fourth most commonly prescribed psychotropic drugs worldwide in adults with ASD^3^. SSRIs are normally prescribed for mood and anxiety disorders, which are highly prevalent (26–57% and 29–54%, respectively^4–7^) in ASD. For this practice is, to the best of our knowledge, currently no available evidence. There is, however, a small body of work suggesting that SSRIs are effective in treating core symptoms in adults with ASD. For instance, two studies of fluoxetine reported significant reductions in repetitive behaviours^8,9^. One study of fluvoxamine in adults with ASD also reported a reduction in repetitive behaviours as well as improvement of social communication^10^. However, these studies included limited sample sizes and the reported benefits were small in comparison to placebo. This indicates that clinical trials are needed to further investigate the effectiveness of SSRIs in adults with ASD, especially as there are currently no pharmacological treatments approved by the Food & Drug Administration (FDA) for treating core symptoms. However, clinical trials have become increasingly expensive^11^, most trials in neuropsychiatric disorders fail, and so industry is investing less in neuroscience. Therefore, it is crucial that we first provide ‘proof of concept’ that potential treatments (e.g. SSRIs) can impact on abnormalities in brain functions associated with core symptoms. Moreover, it has been proposed that core symptoms are partially underpinned by deficits in executive functioning (EF) in ASD^12,13^. Therefore, EF networks may provide novel treatment targets and/or an early ‘read out’ of potential efficacy.

Executive functioning comprises a range of cognitive processes that are required when concentrating and paying and/or switching attention^14^. Individuals with ASD have been reported to have difficulties in performing EF tasks. For example, poorer performance has been reported in adults with ASD compared to controls during performance of a response inhibition task^15^ and in children and adults during performance of sustained attention tasks^16,17^. Additionally, functional MRI studies have demonstrated abnormal brain activation in children and adults with ASD as compared to controls during response inhibition^18,19^ and sustained attention tasks^16,20^. The biological basis of this is poorly understood but may include the serotonergic system, which is involved in motor inhibition^21^ as well as sustained attention^22^. For example, it has been reported in typical developing populations that increasing brain 5-HT with SSRIs can improve performance of a response inhibition task^23^. Studies of sustained attention have also implicated 5-HT—possibly through its dampening effect on noradrenaline, dopamine and acetylcholine, which all play a key role in maintaining high levels of vigilance^24,25^. This is in line with evidence that both single and repeated dosages of SSRIs impair performance during sustained attention tasks in neurotypical populations^22,25–27^.

The serotonergic system is crucial to brain development in embryonic life^28^ and is also implicated in the pathophysiology of ASD. For example, the serotonin (5-HT) transporter gene (SLC6A4) has been associated with ASD^29^, hyperserotonemia is frequently reported in children and adults^30^ and a decrease in density of 5-HT receptors^31^ and the 5-HT transporter^32^ has been observed in adults with ASD. In addition, we previously demonstrated that, in ASD boys, abnormalities in the activation of prefrontal regions during EF tasks (of motor inhibition, cognitive flexibility and working memory) were ‘normalised’ after a single dose of fluoxetine^18,33,34^. However, to date no one has investigated the effect of an SSRI on EF networks in adults with ASD. In addition, no one has tested whether shifting of brain activation after SSRI administration is related to biological serotonergic markers, including peripheral 5-HT levels. If successful, this may help provide a rationale for further testing to determine if modulation of brain function is maintained by longer-term treatment.

Therefore, we tested the effect of the SSRI citalopram on two EF networks (of response inhibition and sustained attention) in adults with ASD using pharmacological functional Magnetic Resonance Imaging (fMRI). Based on our previous work^18,33,34^, we hypothesised that citalopram would modulate abnormal brain activation during EF towards control levels. In addition, we related peripheral 5-HT levels to change in brain activation after citalopram administration. Our subsidiary hypothesis was that degree of responsivity to citalopram would be associated with increased peripheral 5-HT levels.

## Materials and methods

### Participants

Nineteen male, right-handed adults with ASD and 19 typically developed (TD) control participants were included in the study (age: TD mean = 27, SD = 9, ASD mean = 30, SD = 11). Two TD and 2 ASD cases were excluded from the Go/No-Go task due to significant head movement. The sample size was chosen based on results from our prior experiments targeting 5-HT modulation using both acute tryptophan depletion^35,36^, which were successful in detecting group differences in BOLD response with sample sizes of *n* = 14. This implies an effects size (expressed in Cohen’s d) in excess of 1.2^35,36^. Exclusion criteria included medical disorders that could influence cognitive performance, major mental illnesses other than ASD, genetic disorders associated with ASD, alcohol or substance dependence or taking any medication affecting the serotonergic system (e.g. antidepressants, antipsychotics, benzodiazepines or mood stabilisers). ASD diagnosis was made by a consultant psychiatrists using ICD-10 research criteria^1^ and confirmed using the Autism Diagnostic Interview-Revised (ADI-R)^37^ (Communication: mean = 20, SD = 7, Social Interaction: mean = 16, SD = 7, Repetitive Behaviour: mean = 5, SD = 2) if an informant was available. Current autistic symptoms were measured by the Autism Diagnostic Observation Schedule (ADOS)^38^ (Communication: mean = 3, SD = 2, Social Interaction: mean = 6, SD = 2). Intelligence was measured by the Wechsler Abbreviated Scale of Intelligence test (WASI)^39^ (TD mean = 115, SD = 10, ASD mean = 113, SD = 14). All participants completed baseline self-reported questionnaires of autistic traits (Autism-Spectrum Quotient)^40^, obsessionality (Obsessive-Compulsive Inventory-Revised)^41^, and current symptoms of ADHD (Barkley Adult ADHD Rating Scale—IV)^42^. A brief interview was conducted by a medical doctor to assess symptoms of anxiety and depression using The Hamilton Rating Scales for Depression^43^ and Anxiety^44^. All participants gave written, informed consent after receiving a complete description of the study. The study was ethically approved by the Stanmore Ethics Committee.

### Platelet rich plasma serotonin

In order to assess the peripheral serotonergic status of each participant, 7 ml of blood was collected in an anticoagulated EDTA tube, on one occasion, prior to drug administration. One tube was centrifuged at 140 *×* *g* for 25 minutes at 4 °C within 20 min after venipuncture. Centrifugation usually yielded ~3–4 ml of supernatant (platelet-rich plasma, PRP). A 1 ml aliquot of PRP was transferred into a 1.5 ml Eppendorf tube, frozen immediately, and stored at −80 °C until performance of Enzyme-Linked Immunosorbent Assay (ELISA) analysis of plasma 5-HT levels.

### Citalopram administration procedure

For the fMRI scans participants were required to complete two scanning sessions: one after receiving a single dose of 20 mg of encapsulated citalopram and one after receiving a dose of encapsulated placebo (ascorbic acid), in a randomised, double-blind, crossover design. A list of blinding numbers were produced independently and passed directly to the pharmacy in the outpatient department of the Maudsley Hospital using a computerised random number generator and using a blocked randomisation which allowed us to analyse our data at several time points in the study. The pharmacy used these numbers to blind each dose (placebo; encapsulated ascorbic acid/medication; citalopram) as they are manufactured. Both subject and researcher(s) were blind to dosing during the study. The randomisation and encapsulation was conducted according to Good Medical Practice and in accordance with CONSORT & SPIRIT guidelines. Each dose was given to the participant 3 h prior to scanning, as citalopram reaches its peak plasma level after approximately 3 h^45^. There was a minimum of eight days between the scans to allow for complete washout of the drug (t½ = 36 h; washout = 5*t½ = 7.5 days). All participants received a screening by a medical doctor before and after the administration of both doses.

### Visual analogue scale

All participants administered self-reported visual analogue scale questionnaires prior to drug administration and after the fMRI-scan. Symptoms potentially associated with citalopram were measured, including palpitations, nausea, dizziness, attentiveness, anxiety and irritability.

### Go/No-Go inhibition fMRI task

In order to probe the brain’s response inhibition system, a Go/No-Go task was performed during each scanning session^19,46^. During this task, either a motor response on a button box to Go signals is required or the inhibition of this response to No-Go signals. Arrows pointing to either the left or right side appear on the screen. The subject has to press the left or right response button on a diamond-shaped keypad as response. Infrequently (12%), arrows pointing to the top (No-Go signals) appear. Subjects have to inhibit their motor response to these stimuli. In 12% of trials, slightly slanted arrows pointing left or right appear and subjects have to treat them the same way as Go signals. In order to control for the attentional oddball effect due to the low frequency occurrence of No-Go trials, No-Go trials were compared to successful oddball trials^19,46^.

### Sustained attention fMRI task

In order to probe the brain’s sustained attention network system, the sustained attention task was performed during each scanning session^16,20,47^. Subjects need to respond via a right hand button response as quickly as possible, and within 1 s, to the appearance of a visual timer counting up in milliseconds. When they press the button to the counter the counter will show their reaction time as it counts milliseconds. They are instructed to press as soon as they see the counter. The visual stimuli appear either after short, predictable consecutive delays of 0.5 s (260 stimuli in total), in series of 3–5 consecutive stimuli or after unpredictable time delays of 2, 5 or 8 s (20 each), which are pseudo-randomly interspersed into the blocks of 3–5 delays of 0.5 s. The long, infrequent, unpredictable delays place a higher load on sustained attention, as subjects have to wait for them to occur and they do not know the exact time of when they will occur (2, 5 or 8 s) whereas the short, predictable 0.5 s delays which appear in a row are typically anticipated. Participants learn to estimate the 0.5 s and know that there will be several stimuli appearing in a row^48^, placing a higher demand on sensorimotor synchronisation^20^. Activation to the longest delay (8 s) that placed the highest load on sustained attention, and showed most activation, was compared to brain activation during the short delays (0.5 s) (Supplementary Table 1).

We have shown consistently with this task that sustained attention networks are activated during the long delays relative to the short delays and that, with longer delays, there is progressively increasing activation from 2 s to 8 s^16,20,47^.

### Characteristics and performance statistical analysis

Statistical tests were performed using SPSS software (v23.0). *T*-tests were used to compare baseline characteristics between groups and multivariate analysis of variance (MANOVA) to determine any differences in performance and visual analogue scale outcome measures between group and drug conditions. Analysis of variance (ANOVA) was used to compare largest displacement in head movement between group and drug conditions.

For the Go/No-Go task the performance measures include: probability of inhibition (main inhibitory measure), mean reaction time to the Go signal (motor execution measure) and mean reaction time to the oddball signal. For the sustained attention task the performance measures include: coefficient of variation (variation in reaction time during performance of the task adjusted for reaction time, i.e. standard deviation of reaction time divided by reaction time), mean reaction time, premature responses and omission errors.

### fMRI image acquisition

All participants were scanned on a 3-Tesla General Electric Signa HD x Twinspeed scanner (Milwaukee, Wisc.), fitted with a quadrature birdcage head coil. For the fMRI, we acquired 260 (Go/No-Go task) and 480 (sustained attention task) T2*-weighted volumes on 37 (Go/No-Go task) and 31 (sustained attention task) non-adjacent planes parallel to the anterior-posterior commissure (TE = 30 ms, TR = 1.8 s (Go/No-Go task); 1.5 s (sustained attention task), flip angle = 73° (Go/No-Go task); 68° (sustained attention task), slice thickness = 3.0 mm, in-plane voxel-size = 3.75 mm^2^, slice gap = 0.7 mm (Go/No-Go task); 1.4 mm (sustained attention task); matrix size = 64 × 64 voxels). Also, a high resolution gradient echo structural scan, on which activation maps were superimposed, was sagitally acquired to be used during normalisation of the fMRI data into Talairach space. (TR = 3 s, TE = 30 ms, 43 slices, flip angle = 90°, slice gap = 0.3 mm, slice thickness = 3.0 mm, matrix size = 128 × 128 voxels).

### fMRI image analysis

The fMRI data was analysed using XBAM (version 4) software developed at the Institute of Psychiatry, Psychology and Neuroscience^49^. This method is decribed in brief in this section and in more detail in the supplementary material section. This non-parametric approach minimises assumptions involved in image processing and has been previously described^36^. Within each run, every volume was realigned to the mean of all the images in the run and then smoothed using a Gaussian filter (full-width at half-maximum 8.8 mm). Using a wavelet-based resampling method for functional MRI data, a time series analysis was conducted on each individual subject, in order to compute a sum of squares (SSQ) ratio reflecting the BOLD effect. SSQ ratio maps were transformed into standard stereotactic space^50^ using a two-stage warping procedure^49^. First, an average image intensity map for each individual was computed. We then determined the transformations required to map this image to the structural scan for each individual and then from ‘structural space’ to the Talairach template by maximising the correlation between the images at each stage. The SSQ ratios were then transformed into Talairach space using these same transformations. Group brain activation maps were computed for each drug condition with hypothesis testing performed at both the voxel and the cluster level. Using data-driven, permutation-based methods, with minimal distributional assumptions, we performed time series analyses for group maps and inter-group random permutation for within/between-group ANOVAs to compute the distribution of the SSQ ratio under the relevant null distribution hypothesis. Thresholding to the required level of significance was then performed using a two-stage process: first at a voxel-wise *p*-value of 0.05, followed by grouping the supra-threshold voxels into 3D clusters and testing their significance against a null distribution of clusters occurring by chance in the permuted data. A group brain activation map was produced for each group (TD, ASD) and medication (placebo, citalopram) status. We conducted all ANOVA analyses with *p* < 0.05 for voxel level and with *p* < 0.02 at cluster level. This resulted in less than one false positive cluster per map for the sustained attention task and three potentially false positive clusters for the Go/No-Go task.

### Between group analysis of variance

A main effect of group analysis was conducted for the placebo condition for each task. To investigate whether brain activation differences in the ASD group relative to the control group under placebo changed after citalopram dose in ASD, a main effect of group analysis was conducted in regions showing a main effect of group under placebo, but now comparing the control group on placebo with the ASD group on citalopram, to test for potential ‘normalisation’ effects. Furthermore, a within ASD effect of drug analysis was conducted, in regions showing a main effect of group, to investigate whether the degree of change in activation in ASD following citalopram was significant (‘normalised’).

### Group x medication interaction analysis of variance

A two-group (TD, ASD) by two-drug status (placebo, citalopram) factorial repeated-measures ANOVA was conducted for each task. In the groupxdrug status interactions the effect on the BOLD response in brain regions is different in each group depending on drug status. The cluster-level threshold was adjusted to *p* < 0.02, resulting in less than one false-positive cluster per map.

### Correlations between symptomatology, serotonin and change in functional activations

Pearson’s correlations were conducted in XBAM to investigate any associations between core symptoms (measured by the ADI-R and ADOS), associated symptoms of ADHD, anxiety, depression and obsessionality (measured by the BAARS-IV, HAM-A, HAM-D and OCI-R), PRP 5-HT levels and difference in BOLD response between groups under the placebo condition or change between citalopram and placebo conditions (citalopram–placebo), in regions showing a main effect of group during placebo, during both tasks. The sum of square ratio was extracted for each cluster showing a correlation and plotted versus symptomatology or PRP 5-HT levels. A False Discovery Rate analysis was conducted to correct for multiple comparisons.

## Results

### Baseline characteristics

The groups did not significantly differ in age and IQ. As expected, control subjects scored significantly lower on baseline autistic traits and symptoms of anxiety, obsessionality, depression, inattention (childhood) and hyperactivity (currently and in childhood). There was no significant difference between groups in inattention scores currently (Table 1).

**Table 1:** Subjects characteristics

	TD (*n* = 19^a^)	ASD (*n* = 19^a^)	*t-*test *p*-value
Age	27 ± 9 (19–52)	30 ± 11 (19–50)	0.3
IQ	115 ± 10 (88–130)	113 ± 14 (79–139)	0.7
ADOS–Communication	–	3 ± 2	
ADOS–Social interaction	–	6 ± 2	
ADI-R–Communication	–	17 ± 9	
ADI-R–Social interaction	–	14 ± 8	
ADI-R–Repetitive behaviour	–	5 ± 2	
AQ	12 ± 7	31 ± 11	<0.001***
HAM-D	2 ± 3	6 ± 4	0.001**
HAM-A	3 ± 4	8 ± 6	0.003**
OCI-R	8 ± 9	23 ± 13	<0.001***
GAD-7	3 ± 3	7 ± 5	0.01*
Barkley inattention childhood self	0.7 ± 1.2	3.3 ± 3.0	0.002**
Barkley hyperactivity childhood self	1.1 ± 1.9	3.7 ± 2.9	0.004**
Barkley inattention currently self	0.7 ± 1.5	1.4 ± 1.8	0.2
Barkley hyperactivity currently self	0.4 ± 0.8	1.2 ± 1.3	0.004**

Data in table is shown as mean ± standard deviation (range) (*n* = number of participants)

*TD* typically developed controls, *ASD* individuals with autism spectrum disorder, *ADOS* Autism diagnostic observation scale, *ADI-R* Autism diagnostic interview-revised, *AQ* Autism quotient, *HAM-D* Hamilton Depression Rating Scale, *HAM-A* Hamilton Anxiety Rating Scale, *OCI-R* obsessive-compulsive inventory revised

Between group *t*-test: * = *p* < 0.05; ** = *p* < 0.01; *** = *p* < 0.001

^a^*n* = 19 for Sustained Attention task and *n* = 17 for Go/No-Go task, which did not significantly affect between-group differences in baseline characteristics, platelet rich plasma serotonin (PRP 5-HT) levels or visual analogue scale measures

### Platelet rich plasma serotonin levels

The data was normally distributed for each group, as assessed by the Shapiro-Wilk test (*p* > 0.05). No significant differences were observed between groups in mean PRP 5-HT levels (TD mean = 324.1 μg/l, SD = 220.2, ASD mean = 316.1 μg/l, SD = 168.3; *t*(36) = 0.11, *p* *=* 0.91).

### Performance

#### Go/No-Go task

Multivariate analysis of variance revealed no significant between-group or within-group differences for the probability of inhibition or mean reaction time to the Go or oddball stimuli (Supplementary Table 2).

#### Sustained attention task

Multivariate analysis of variance revealed significant differences between TD and ASD during both the placebo and citalopram conditions on mean reaction time and coefficient of variation for the 0.5 and 8 s delays. Mean reaction time was slower and coefficient of variation higher in ASD compared to controls. For omission errors there was a significant group difference during the placebo condition for the 0.5 s delay and for premature responses, there were significant group differences during both placebo and citalopram conditions for the 0.5 s delay, with ASD showing more omission errors and premature responses. There were no significant within group differences in performance outcome following citalopram in both groups. However, when comparing control subjects during placebo with ASD cases during citalopram the group difference of coefficient of variation for the 8 s delay condition was no longer significant (Supplementary Table 3).

#### Visual analogue scales

Despite baseline group differences in associated symptomatology (Table 1), multivariate analysis of variance showed no significant difference after placebo or citalopram intake in both groups on subjective reports of physical and psychological symptoms, including palpitations, nausea, dizziness, anxiety, depression or irritability (Supplementary Table 4).

#### Movement

##### Go/No-Go task

For largest head displacement in three-dimensional space there was no significant effect of group (*F*(1, 64) = 1.13; *p* = 0.29), drug (*F*(1, 64) = 2.56; *p* = 0.11) or group × drug interaction (*F*(1, 64) = 0.02; *p* = 0.89) (Supplementary Table 5).

##### Sustained attention task

For largest head displacement in three-dimensional space there was no significant effect of group (*F*(1, 72) = 2.3; *p* = 0.14), drug (*F*(1, 72) = 1.07; *p* = 0.30) or group × drug interaction (*F*(1, 72) = 0.01; *p* = 0.92) (Supplementary Table 5).

##### Within group brain activations

The group activation maps for each group (TD, ASD) and medication (placebo, citalopram) status revealed significant activation during successful inhibition (No-Go > Oddball) in response inhibition regions including inferior, superior and middle frontal, and pre- and postcentral cortex and cerebellum. During sustained attention significant activation was observed in superior and middle frontal, superior and middle temporal, occipital and pre- and postcentral cortices and cerebellum (Supplementary Tables 6–13, Supplementary Figs. 1 and 2).

### Group differences in brain activation during placebo and citalopram

#### Go/No-Go task

**Fig. 1:**
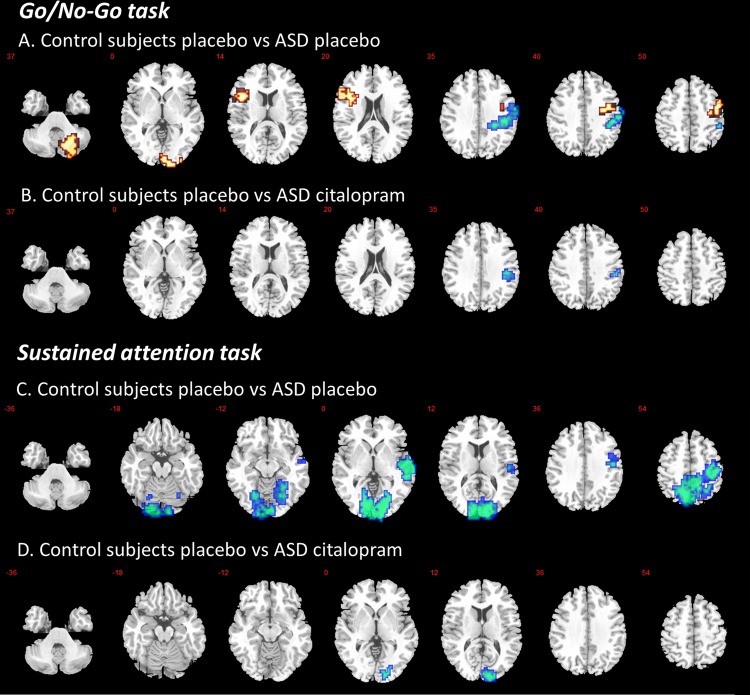
Brain activation map showing abnormally activated regions during response inhibition and sustained attention in ASD that were ‘normalised’ by citalopram; *p* < 0.02 at cluster level. Location of BOLD signal changes between groups. Red: TD < ASD; Blue: TD > ASD. Numeric label = z Talairach coordinate. Right hemisphere of brain is on the right side of the image. Abbreviations: BOLD, blood-oxygen-level dependent; TD, Typically Developed Controls; ASD, Individuals with Autism Spectrum Disorder

During placebo, subjects with ASD relative to controls showed a decrease in activation in right postcentral cortex (*p* = 0.009, cluster size = 144 voxels). An increase in activation in ASD compared to controls was observed in left inferior frontal cortex/left insula (*p* = 0.02, cluster size = 95 voxels), right precentral cortex (*p* = 0.01, cluster size = 111 voxels), right cerebellum (*p* = 0.02, cluster size = 81 voxels) and right occipital cortex (*p* = 0.01, cluster size = 114 voxels) (Fig. 1a, Table 2).

**Table 2:** Anatomical location and statistics for BOLD activation

Region	*X*	*Y*	*Z*	Cluster *p*-value	Cluster size
*GO/NO-GO task*					
*TD placebo vs ASD placebo*					
*TD>ASD (blue)*					
Right postcentral cortex	58	−19	33	0.009	144
*TD<ASD (red)*					
Right cerebellum	29	−67	−40	0.02	81
Right occipital cortex	11	−96	−7	0.01	114
Left inferior frontal cortex/left insula	−40	19	13	0.02	95
Right precentral cortex	43	−7	50	0.01	111
*TD placebo vs ASD citalopram*					
*TD>ASD (blue)*					
Right postcentral cortex	43	−22	33	0.005	25
Interaction of drug status (placebo, citalopram) by group (TD, ASD)					
Left Cerebellum, Posterior Lobe (Crus I/II)	−22	−81	−33	0.01	132
*SUSTAINED ATTENTION task*					
*TD placebo vs ASD placebo*					
*TD>ASD (blue)*					
Right middle temporal cortex	61	−7	−7	0.02	212
Right cuneus	14	−93	3	0.0008	1082
Left precuneus	−4	−63	46	0.009	747
*TD placebo vs ASD citalopram*					
*TD>ASD (blue)*					
Right Cuneus	14	−93	3	0.03	129

x, y, z = Peak Talairach coordinates

*BOLD* blood-oxygen-level dependent, *TD* typically developed controls, *ASD* individuals with Autism spectrum disorder

**Fig. 2:**
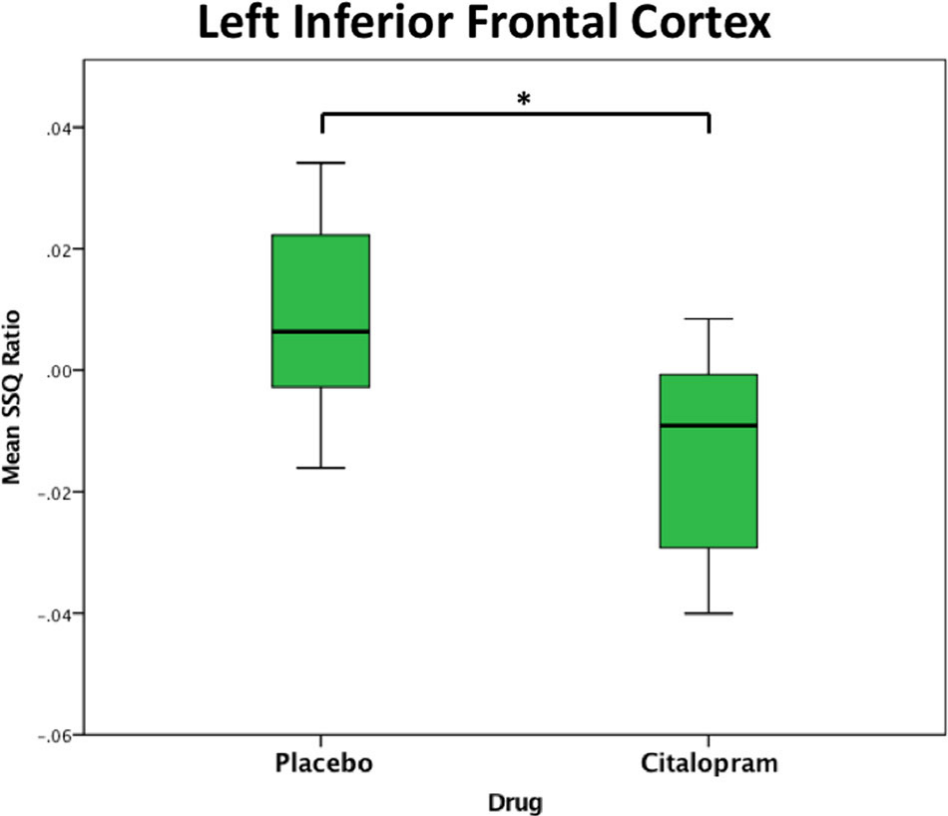
Significant decrease (‘normalisation’) in brain activation during response inhibition in left inferior frontal cortex within ASD following citalopram administration. Abbreviations: SSQ, sum of squares (statistical measure of BOLD response); * = *p* < 0.05

In order to test potential ‘normalisation’ effects, subjects with ASD during citalopram were compared to controls during placebo focusing on regions where between-group differences under placebo were observed. Nearly all between group differences were no longer observed, leaving only one small decrease in activation in subjects with ASD compared to controls in right postcentral cortex (*p* = 0.005, cluster size = 25 voxels) (Fig. 1b, Table 2). Subsequently, a within ASD analysis was conducted to investigate the effect of drug in those regions specifically. Only the decrease in overactivation of left insula significantly ‘normalised’ (*p* = 0.04, see Fig. 2).

#### Sustained attention task

During placebo, subjects with ASD relative to controls showed a decrease in activation in right middle temporal cortex (*p* = 0.02, cluster size = 212 voxels), right cuneus (*p* = 0.001, cluster size = 1082 voxels) and left precuneus (*p* = 0.009, cluster size = 747 voxels) (Fig. 1c, Table 2).

In order to test potential ‘normalisation’ effects, subjects with ASD during citalopram were compared to controls during placebo focusing on regions where between-group differences under placebo were observed. Nearly all between group differences were no longer observed, leaving only one small decrease in activation in subjects with ASD compared to controls in right cuneus (*p* = 0.03, cluster size = 129 voxels) (Fig. 1d, Table 2). Subsequently, a within ASD analysis was conducted to investigate the effect of drug in those regions specifically. Non of the regions ‘normalised’ significantly.

#### Group by drug interaction effects

##### Go/No-Go task

**Fig. 3:**
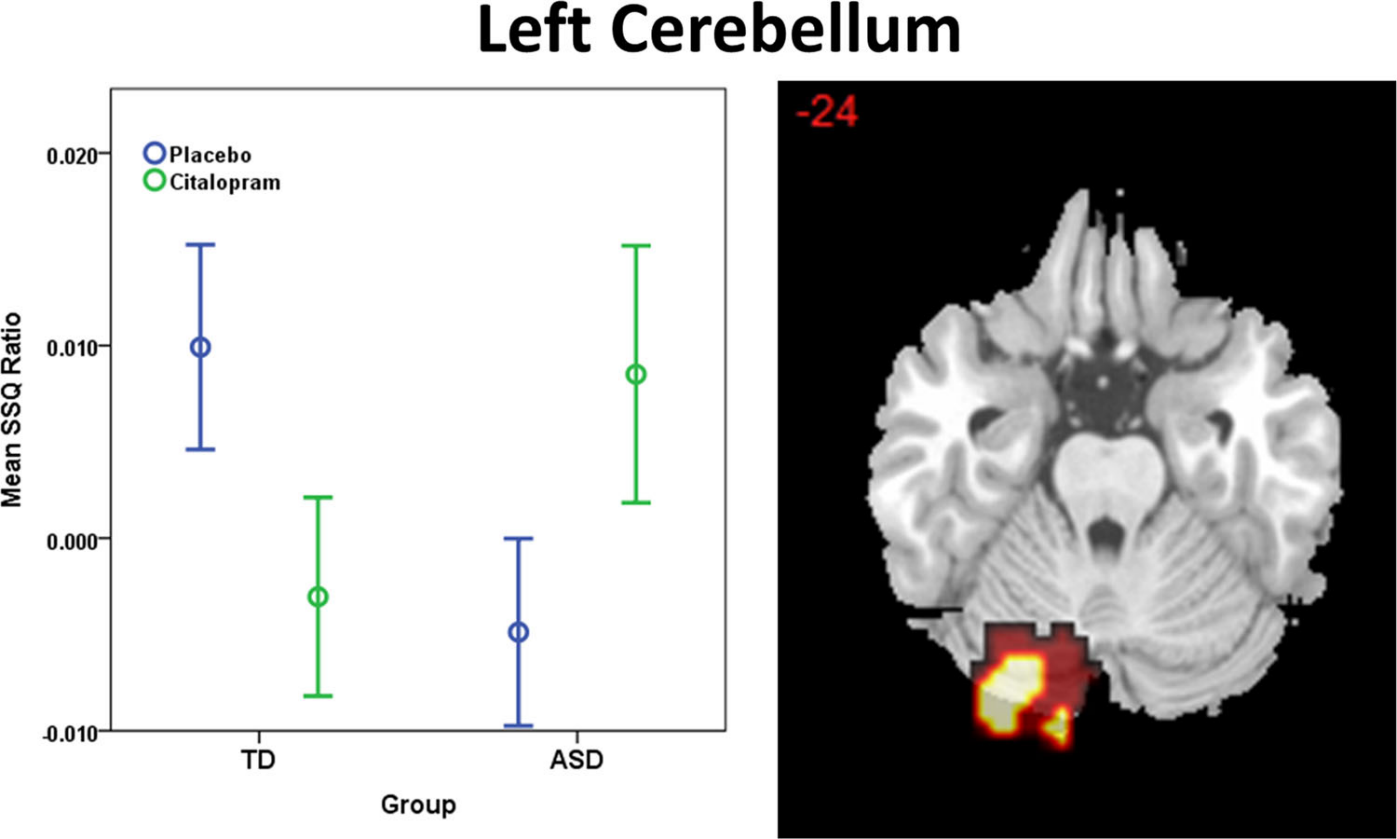
Interaction of drug status (placebo, citalopram) by group (TD, ASD) during response inhibition; *p* < 0.02 at cluster level. Location of BOLD signal for ANOVA interaction. Numeric label = z Talairach coordinate. Box plots: Mean BOLD signal extracted from each interaction cluster. Right hemisphere of brain is on the right side of the image. Abbreviations: SSQ, sum of squares fMRI statistic; BOLD, blood-oxygen-level dependent; TD, Typically Developed Controls; ASD, Individuals with Autism Spectrum Disorder

There was a significant interaction effect of BOLD signal response between drug status (placebo, citalopram) and group (TD, ASD) in left cerebellum (posterior lobe, crus I/II; *p* = 0.01 cluster size = 132 voxels). In this region citalopram decreased brain activation in the control group but increased brain activation in ASD (Fig. 3 and Table 2).

##### Sustained attention task

There were no significant interaction effects of BOLD signal response between drug status (placebo, citalopram) and group (TD, ASD).

### Correlations between functional activations and symptomatology and peripheral 5-HT within ASD

#### Go/No-Go task

Within ASD, baseline PRP 5-HT levels correlated positively with BOLD signal under placebo condition in the left insula (*r* = 0.81, *p* < 0.001, cluster size = 23; at baseline increased activation in ASD compared to TD; extending from left inferior frontal cortex). Also, the degree of BOLD signal change between citalopram and placebo correlated negatively with PRP 5-HT levels in left insula (*r* = −0.8, *p* < 0.0001, cluster size = 42 voxels; at baseline increased activation in ASD compared to TD; extending from left inferior frontal cortex) and positively in the right postcentral cortex (*r* = 0.8, *p* < 0.0001, cluster size = 52 voxels; at baseline decreased activation in ASD compared to TD) (Supplementary Fig. 3). Hence, the higher the individual’s PRP 5-HT levels were at baseline, the more likely their brain activation in left insula and right postcentral cortex would ‘normalise’ after citalopram.

#### Sustained attention task

Within ASD, ADOS communication scores correlated negatively with BOLD signal under placebo condition in right cuneus (*r* = −0.59, *p* = 0.01, cluster size = 201; at baseline decreased activation in ASD compared to TD). Furthermore, the degree of BOLD signal change between citalopram and placebo correlated positively with severity of current hyperactivity symptoms in the right middle temporal cortex (*r* = 0.7, *p* = 0.001, cluster size = 71 voxels; at baseline decreased activation in ASD compared to TD) and with severity of current inattention symptoms in the right postcentral cortex (*r* = 0.7, *p* = 0.001, cluster size = 84 voxels; at baseline decreased activation in ASD compared to TD) (Supplementary Fig. 3). Hence, the more severe an individual’s hyperactivity or inattention scores were at baseline, the more likely their brain activation would ‘normalise’ after citalopram.

No correlations between functional activations and PRP 5-HT levels or symptoms were observed in the TD group.

## Discussion

To our knowledge, this is the first fMRI study to examine the effect of citalopram on brain activation during EF in adults with ASD. We observed abnormal brain activation in adults with ASD compared to controls during successful inhibitory control in left inferior frontal, right precentral and postcentral cortices, and right cerebellum. During sustained attention, we observed reduced brain activation in ASD compared to controls in the right middle temporal cortex, right cuneus and left precuneus. Following citalopram dosing, these atypical brain activations during both tasks were mostly ‘abolished’ and during response inhibition ‘normalised’ in left inferior frontal cortex. Further studies are needed to determine if ‘normalisation’ of brain response during EF tasks is maintained by longer term citalopram treatment, and associated with treatment response.

The observed group differences under placebo condition in brain activation during the EF tasks are partially in line with previous work. For instance, we previously reported abnormal brain activation during response inhibition in children with ASD^18^ and adults with Asperger’s syndrome^19^. Both studies reported increased activation of left inferior frontal cortex, a region that is critical for successful response inhibition, specifically motor responses^51^. Bilateral inferior frontal cortex has been associated with inhibitory control in fMRI^52–54^, lesion^55^ and transcranial magnetic stimulation (TMS) studies^56,57^. However, bilateral inferior frontal cortex has also been associated with the broader role in target detection or oddball attention processes that may expedite response inhibition^58–60^. Likewise, the finding in the present study of decreased brain activation during sustained attention partially overlaps with our previous studies of the same task in ASD children^20^ and a mixture of children and adults^16^. In these studies we reported decreased brain activity in prefrontal, parietal, temporal, striato-thalamic and cerebellar regions. Thus, our findings of abnormal brain activation during both tasks support the suggestion that individuals with ASD have abnormalities in brain activation during EF.

Our finding of ‘normalisation’ in brain function differences during response inhibition after citalopram is also in line with a previous fMRI investigation of an acute dose of fluoxetine in boys with ASD^18^. Taken together these studies suggest that SSRIs can modulate abnormal brain activation in both children and adults with ASD. In addition, within ASD individuals we observed a strong association between PRP 5-HT levels and the degree of ‘normalisation’ in left inferior frontal cortex and right postcentral cortex. In contrast, however, no associations were observed within the control group. This suggests that baseline PRP 5-HT levels may underpin brain functional response to citalopram, but specifically in ASD. However, as we did not include a control group with a mood or anxiety disorder, we cannot rule out the possibility that this effect may also be present in such populations.

The ‘abolition’ effect of citalopram on brain function abnormalities in sustained attention networks in ASD is a novel finding. Also, the degree of ‘abolition’ after citalopram in right middle temporal and postcentral cortex correlated with hyperactivity and inattention symptoms, respectively. In addition, impaired task performance in ASD compared to controls was no longer significant after citalopram administration (for coefficient of variation). Moreover, we observed a significant increase in a self-reported measure of inattentiveness after citalopram in the control group, but not in ASD. This suggests that citalopram improves both attention performance and its underlying neurofunctional networks in ASD. This could potentially transfer into clinical improvement of inattention symptoms, which are also present in frequent comorbid psychiatric conditions such as ADHD^61^ and depression^62,63^.

Our interaction analysis during response inhibition revealed that in the left cerebellum (posterior lobe crus I/II), a region that is involved in cognitive control^64,65^ and connected to the frontal cortex^66^, citalopram shifted brain activation in the opposite direction in ASD compared to controls - citalopram decreased brain activation in the control group, but increased brain activation in ASD. This “reversal” of brain activation may reflect structural cerebellar abnormalities as well as dysfunctions of frontal-cerebellar networks that have frequently been reported in ASD populations. For example, abnormalities in structural white matter connectivity both within the cerebellum as well as its cortical and mid-brain projections have been reported in ASD^67,68^. Functional connectivity studies support these findings, suggesting differences in connections between the cerebellum and both motor and non-motor regions of the cortex in ASD^69,70^. This suggests that the neuropharmacological impact of citalopram is different in ASD than in controls. Therefore, it is possible that results from treatment studies in typically developed populations cannot be directly translated to individuals with ASD. This stresses the need for pharmacological interventions, affecting the serotonergic system, to be tested specifically in ASD.

Our study has a number of limitations. First, we only included high functioning adult males and therefore cannot generalise our findings to other groups including females, children or lower functioning individuals. Second, we only used a single dosage of citalopram and we therefore cannot comment on whether the ‘normalisation’ effects will be maintained by long-term treatments. Third, for the Go/No-Go task, we did not find any differences in performance measures. This, however, is in line with a previous study using the Go/No-Go task^35^ and possibly caused by our relatively small sample size, that may be underpowered for behavioural data. For example, a recent study using a much larger sample (201 ASD cases and 240 controls) employed an online Go/No-Go task and reported deficits in response inhibition that was associated with diagnosis and autistic traits^15^. Nevertheless, our sample was large enough to detect brain activation differences, which have previously been reported to be more sensitive to drug effects than behaviour, including in fMRI studies of ASD^34,35^. Last, the *p*-value at cluster level was set at 0.02, which resulted in less than one false positive cluster per map for the sustained attention task but three potentially false positive clusters for the Go/No-Go task. Therefore, three of the reported regions may be false-positive clusters. However, we decided to include them as they overlap with regions previously reported to show abnormal activation in ASD population (e.g. increased activation in left inferior frontal cortex)^18,19,35^.

In conclusion, we report that, within individuals with ASD, citalopram can ‘normalise’ brain functional differences during EF, and these modulations are associated with PRP 5-HT levels. This suggests a potential utility of citalopram for targeting EF related problems (e.g. core symptoms) in ASD. Therefore, future trials should investigate whether this shift in brain activation after a single dosage of citalopram is maintained after prolonged treatment, and whether this is associated with treatment response. Additionally, further studies are required to determine whether long-term treatment outcome to SSRIs is associated with elevated 5-HT levels.

## Supplementary information

Supplementary material
