# Supplementary material for: Modulation of brain activation during executive functioning in autism with citalopram
Source: Transl Psychiatry. 2019 Nov 11;9:286. doi: 10.1038/s41398-019-0641-0 (PMC6848075; doi:10.1038/s41398-019-0641-0)
Supplement: Supplementary file 1 — Supplementary material [file 41398_2019_641_MOESM1_ESM.docx]

# Supplementary information

## fMRI preprocessing

Data were analyzed with version 4.1 of the XBAM software developed at the King’s College London Institute of Psychiatry, Psychology & Neuroscience, (using a non-parametric approach (for a full description and references, see **www.brainmap.co.uk**)^1^. XBAM uses median statistics to control for outlier effects and employs permutation rather than normal theory based inference. Furthermore, its most common test statistic (sum of squares ratio [SSQ]) is computed by standardising for individual difference in residual noise before embarking on second level, multi-subject testing using robust permutation-based methods. This allows a mixed effects approach to analysis to deal with the issue of non-normality. The use of a mixed effect approach addresses the issue of inequality of individual residual variances by effectively “down weighting” responses with large residual variances. The significance of the resulting reweighted responses at group level is then tested by data permutation to avoid assumptions of normality.

## fMRI data analysis XBAM

Data were analyzed with software developed at the Institute of Psychiatry, London, (XBAM) using a non-parametric approach (for a full description and references, see **www.brainmap.co.uk**). XBAM uses median statistics to control for outlier effects and employs permutation rather than normal theory based inference. Furthermore, its most common test statistic (sum of squares ratio [SSQ]) is computed by standardising for individual difference in residual noise before embarking on second level, multi-subject testing using robust permutation-based methods. This allows a mixed effects approach to analysis to deal with the issue of non-normality. The use of a mixed effect approach addresses the issue of inequality of individual residual variances by effectively “down weighting” responses with large residual variances. The significance of the resulting reweighted responses at group level is then tested by data permutation to avoid assumptions of normality.

## Individual Brain Activation Mapping

Within each run, each time point was realigned to the mean of all the images in the run to remove subject-induced motion artifacts.^2^ Following realignment, data were then smoothed using a Gaussian filter (FWHM 8.8 mm) to improve the signal to noise characteristics of the images. Responses to the experimental paradigms were then detected by first convolving each component of the experimental design with each of two gamma variate functions (peak responses at 4 and 8 sec respectively). These two functions were chosen to encompass the known range of times to peak response following stimulus onset for BOLD effects. The best fit between the weighted sum of these convolutions and the time series at each voxel was computed using the constrained BOLD effect model.^3^ Following computation of the model fit, a goodness of fit statistic was computed. This consisted of the ratio of the sum of squares of deviations from the mean image intensity (over the whole time series) due to the model to the sum of squares of deviations due to the residuals (SSQ ratio). Following computation of the observed SSQ ratio at each voxel, the data are permuted (50 permutations at each voxel and then all the permutations compiled together to form a big probability distribution) using a wavelet-based method.^4^ Repeated application of this strategy at each voxel followed by recomputation of the SSQ ratio from the permuted data allows (by combination of results over all intracerebral voxels) the data-driven calculation of the null distribution of SSQ ratios under the assumption of no experimentally determined response. Using this distribution it is possible to calculate the critical value of SSQ ratio needed to threshold the maps at any desired type I error rate. Then, detection of activated voxels was extended from voxel to cluster level. ^5^

## Group Brain Activation Mapping

The observed and permuted SSQ ratio maps for each individual were transformed into the standard space ^6^ using a two stage warping procedure.^7^ For both stages of the warping process, a twelve parameter affine transform was employed. This involves first computing the average image intensity map for each individual over the course of the experiment. The transformations required to map this image to the structural scan for each individual and then from “structural space” to the Talairach template were then computed by maximizing the correlation between the images at each stage. The SSQ ratio maps were then transformed into Talairach space using these transformations. Group activation maps were then computed by determining the median SSQ ratio at each voxel (over all individuals) in the observed and permuted data maps (medians are used to minimize outlier effects). The distribution of median SSQ ratios over all intracerebral voxels from the permuted data was then used to derive the null distribution of SSQ ratios and which can then be thresholded to produce group activation maps at any desired voxel or cluster-level type I error rate. In this two level clustering procedure,^5^ the first (voxel-wise) thresholding is carried out at an uncorrected p value of 0.05 to give the maximum allowable sensitivity. In order to eliminate the resulting false positive activations, a second, cluster-level thresholding step is carried out and the threshold of this second step is adjusted to give an expectation of less than one false positive cluster over the whole brain. As the cluster level threshold is set at the whole brain level, the normal, voxel-wise issue of multiple comparisons does not apply. The computation of a standardized measure of effect SSQ ratio at the individual level, followed by analysis of the median SSQ ratio maps over all individuals treats intra- and inter-subject variations in effect separately. This constitutes a mixed-effect approach, which allows for inferences from these results to be made about the larger population.

## Supplementary table 1: Increase in brain activation with higher sustained attention load

| **Delay** | **Total Activated Cluster size** |
| --- | --- |
| **TD placebo condition** |  |
| 2s > 0.5s | 3580 |
| 5s > 0.5s | 5812 |
| 8s > 0.5s | 6609 |
| **ASD placebo condition** |  |
| 2s > 0.5s | 1314 |
| 5s > 0.5s | 4040 |
| 8s > 0.5s | 4806 |

Abbreviations: TD, Typically Developed Controls; ASD,

Individuals with Autism Spectrum Disorder.

## Supplementary table 2: Go/No-Go task performance measures

|  | **TD** |  |  |  | **ASD** |  |  |  | **Difference TD, ASD** | | | |
| --- | --- | --- | --- | --- | --- | --- | --- | --- | --- | --- | --- | --- |
| **Performance measures** | **Placebo** | **Citalopram** | ***p*-value** | ***F* (1, 32)** | **Placebo** | **Citalopram** | ***p*-value** | ***F* (1, 32)** | **Placebo  *p*-value** | ***F* (1, 32)** | **Citalopram *p*-value** | ***F* (1, 32)** |
| P(I) % | 97 ± 4.0 | 96 ± 5.8 | 0.6 | 0.3 | 95 ± 4.3 | 96 ± 5.0 | 0.7 | 0.2 | 0.2 | 1.4 | 1.0 | <0.001 |
| MRT Go (ms) | 463 ± 86 | 468 ± 78 | 0.8 | 0.04 | 463 ± 53 | 459 ± 58 | 0.8 | 0.1 | 1.0 | 0.001 | 0.7 | 0.2 |
| MRT Odd (ms) | 488 ± 96 | 493 ± 84 | 0.9 | 0.2 | 480 ± 51 | 478 ± 57 | 0.9 | 0.01 | 0.8 | 0.1 | 0.6 | 0.3 |

Data in table is shown as mean ± standard deviation. Abbreviations: P(I), probability of inhibition; MRT Go, mean reaction time to Go signal; MRT Odd, mean reaction time to Oddball signal

## Supplementary table 3: Performance measures sustained attention task

| **Performance Measure** | **Delay (s)** | **TD** | | |  | **ASD** | | |  | **Difference TD, ASD** | | | | **TD placebo vs ASD citalopram** | |
| --- | --- | --- | --- | --- | --- | --- | --- | --- | --- | --- | --- | --- | --- | --- | --- |
|  |  | **Placebo** | **Citalopram** | ***p* - value** | ***F***  **(1, 36)** | **Placebo** | **Citalopram** | ***p* - value** | ***F***  **(1, 36)** | **Placebo**  ***p* - value** | ***F***  **(1, 36)** | **Citalopram**  ***p* - value** | ***F***  **(1, 36)** | ***p-*value** | ***F***  **(1, 36)** |
| **Mean reaction time (RT) (ms)** | 0.5s  8s | 310 ± 32  410 ± 52 | 310 ± 33  400 ± 52 | 0.9  0.7 | 0.1  0.2 | 350 ± 55  450 ± 52 | 360 ± 61  460 ± 58 | 0.4  0.5 | 0.6  0.4 | 0.03*  0.03* | 5.4  5.2 | 0.005**  0.003** | 9.1  9.9 | 0.005**  0.01* | 9.6  7.7 |
| **Coefficient of variation (SD/mean of RT) (ms)** | 0.5s  8s | 61 ± 23  59 ± 37 | 64 ± 23  56 ± 33 | 0.7  0.7 | 0.1  0.1 | 82 ± 26  85 ± 35 | 84± 29  78 ± 38 | 0.8  0.6 | 0.1  0.3 | 0.01*  0.04* | 7.1  4.8 | 0.02*  0.06 | 6.1  3.7 | 0.01*  0.13 | 7.6  2.3 |
| **Omission errors** | 0.5s  8s | 1.6 ± 4.8  0.7 ± 1.9 | 4.5 ± 15  0.5 ± 1.1 | 0.4  0.7 | 0.6  0.2 | 9.4 ± 13  1.1 ± 2.5 | 6.2 ± 8.3  0.6 ± 1.1 | 0.4  0.5 | 0.8  0.6 | 0.03*  0.6 | 5.7  0.3 | 0.7  0.7 | 0.2  0.2 | 0.05*  0.92 | 4.4  0.01 |
| **Premature responses** | 0.5s  8s | 0.9 ± 2.0  3.1 ± 3.6 | 0.8 ± 1.8  3.5 ± 3.0 | 0.9  0.7 | 0.3  0.1 | 3.3 ± 4.0  3.4 ± 3.6 | 10.7 ± 17  4.0 ± 4.5 | 0.08  0.6 | 3.3  0.2 | 0.03*  0.8 | 5.4  0.1 | 0.02*  0.7 | 6.2  0.2 | 0.02*  0.51 | 6.0  0.5 |

Data in table is shown as mean ± standard deviation. *=*P*<0.05; **= *P*<0.01; Abbreviations: s, seconds; ms, milliseconds; SD, standard deviation; TD, Typically Developed Controls; ASD, Individuals with Autism Spectrum Disorder; RT, mean reaction time.

## Supplementary table 4: Visual analogue scales (VAS) of self-reported symptoms potentially associated with citalopram

| **Group** | **Drug** | **Time** | **Palpitations** | **Nausea** | **Dizziness** | **Inattentiveness** | **Anxiety** | **Depression** | **Irritability** |
| --- | --- | --- | --- | --- | --- | --- | --- | --- | --- |
| **TD** |  |  |  |  |  |  |  |  |  |
|  | **Placebo** |  |  |  |  |  |  |  |  |
|  |  | Baseline | 10.0 ± 16.4 | 9.8 ± 19.2 | 7.1 ± 17.4 | 24.8 ± 25.1 | 8.6 ± 12.7 | 22.1 ± 19.7 | 8.4 ± 17.1 |
|  |  | 4.5 h post drug intake | 3.5 ± 4.0 | 2.7 ± 3.4 | 3.1 ± 4.4 | 26.5 ± 25.6 | 6.5 ± 5.3 | 17.9 ± 16.0 | 4.7 ± 7.4 |
|  |  | *p*-value | 0.1 | 0.1 | 0.3 | 0.8 | 0.5 | 0.5 | 0.4 |
|  |  | *F* (1, 36) | 2.8 | 2.5 | 0.9 | 0.04 | 0.4 | 0.5 | 0.7 |
|  | **Citalopram** |  |  |  |  |  |  |  |  |
|  |  | Baseline | 6.1 ± 8.3 | 5.5 ± 11.5 | 4.9 ± 9.7 | 17.7 ± 16.7 | 7.5 ± 8.8 | 17.5 ± 14.8 | 7.5 ± 11.9 |
|  |  | 4.5 h post drug intake | 5.7 ± 8.0 | 8.7 ± 14.9 | 5.7 ± 8.3 | 31.1 ± 23.9 | 5.8 ± 6.8 | 18.9 ± 13.7 | 7.7 ± 10.5 |
|  |  | *p*-value | 0.9 | 0.5 | 0.8 | 0.1 | 0.5 | 0.8 | 1.0 |
|  |  | *F* (1, 36) | 0.03 | 0.6 | 0.1 | 3.9 | 0.4 | 0.1 | 0.003 |
| **ASD** |  |  |  |  |  |  |  |  |  |
|  | **Placebo** |  |  |  |  |  |  |  |  |
|  |  | Baseline | 3.9 ± 6.3 | 4.7 ± 6.7 | 5.9 ± 9.6 | 33.5 ± 26.5 | 15.7 ± 14.2 | 26.9 ± 23.4 | 8.0 ± 14.3 |
|  |  | 4.5 h post drug intake | 6.5 ± 11.0 | 4.8 ± 8.3 | 5.3 ± 8.2 | 26.9 ± 24.0 | 12.7 ± 17.1 | 24.8 ± 25.2 | 8.3 ± 14.9 |
|  |  | *p*-value | 0.4 | 1.0 | 0.8 | 0.4 | 0.6 | 0.8 | 0.9 |
|  |  | *F* (1, 35) | 0.8 | 0.002 | 0.1 | 0.6 | 0.3 | 0.1 | 0.004 |
|  | **Citalopram** |  |  |  |  |  |  |  |  |
|  |  | Baseline | 6.4 ± 8.3 | 7.0 ± 11.4 | 14.2 ± 16.6 | 27.0 ± 4.6 | 18.6 ± 22.5 | 28.1 ± 25.7 | 15.2 ± 19.0 |
|  |  | 4.5 h post drug intake | 8.2 ± 11.7 | 10.6 ± 15.3 | 15.7 ± 20.1 | 36.5 ± 28.1 | 13.2 ± 13.1 | 28.9 ± 25.4 | 13.8 ± 18.3 |
|  |  | *p*-value | 0.6 | 0.4 | 0.8 | 0.3 | 0.4 | 0.9 | 0.8 |
|  |  | *F* (1, 36) | 0.3 | 0.7 | 0.1 | 1.2 | 0.8 | 0.01 | 0.1 |

Data in table is shown as mean ± standard deviation. Abbreviations: TD, Typically Developed Controls; ASD, Individuals with Autism Spectrum Disorder.

## Supplementary table 5: Subject Movement

| D statistic | TD placebo | TD citalopram | ASD placebo | ASD citalopram |
| --- | --- | --- | --- | --- |
| Go/No-Go Task | 1.6 ± 0.6 | 2.0 ± 0.8 | 1.8 ± 0.8 | 2.3 ± 1.9 |
| Sustained Attention Task | 2.5 ± 2.2 | 3.2 ± 2.8 | 3.5 ± 2.6 | 4.3 ± 4.2 |

Data in table is shown as mean ± standard deviation. Abbreviations: TD, Typically Developed Controls; ASD, Individuals with Autism Spectrum Disorder. D statistic: D = SQRT ((Largest_displacement_x*Largest_displacement_x) + (Largest_displacement_y*Largest_displacement_y) + (Largest_displacement_z*Largest_displacement_z)); Largest displacement in mm in three dimensions (x, y and z)

***Supplementary table 6:*** *Anatomical location and statistics for BOLD activation for the Go/No-Go task (TD group, placebo condition)*

| **Region** | **X** | **Y** | **Z** | **Cluster *p*-value** | **Cluster size** |
| --- | --- | --- | --- | --- | --- |
| **No-Go < Oddball (blue)** |  | | | | |
| Right Cerebellum | 18 | -67 | -40 | 0.03 | 59 |
| Right Putamen | 22 | 4 | 17 | 0.02 | 59 |
| Right Posterior Cingulate | 7 | -56 | 17 | 0.02 | 107 |
| Right Cuneus | 7 | -85 | 30 | 0.006 | 204 |
| Right Medial Frontal Cortex | 29 | -4 | 36 | 0.05 | 29 |
| Left Posterior Cingulate | -22 | -63 | 10 | 0.02 | 106 |
| Left Inferior Frontal Cortex | -40 | 15 | 13 | 0.03 | 78 |
| Left Superior Frontal Cortex | -11 | 44 | 36 | 0.05 | 25 |
| Left Precentral Cortex | -36 | -22 | 53 | 0.004 | 227 |
| **No-Go > Oddball (red)** |  | | | | |
| Right Occipital Cortex | 11 | -93 | 17 | 0.04 | 35 |
| Right Superior Temporal Cortex | 58 | -52 | 20 | 0.04 | 40 |
| Right Precentral Cortex | 61 | 0 | 26 | 0.04 | 34 |
| Right Postcentral Cortex | 43 | -22 | 33 | 0.03 | 67 |
| Left Cerebellum | -29 | -74 | -20 | 0.04 | 41 |
| Left Middle Temporal Cortex | -47 | -59 | 17 | 0.01 | 125 |
| Left Precuneus | 0 | -44 | 50 | 0.04 | 32 |

x, y, z = Peak Talairach coordinates. Abbreviations: BOLD, blood-oxygen-level dependent; TD, typically developed controls; ASD, individuals with autism spectrum disorder.

***Supplementary Table 7:*** *Anatomical location and statistics for BOLD activation for the Go/No-Go task (TD group, citalopram condition)*

| **Region** | **X** | **Y** | **Z** | **Cluster *p*-value** | **Cluster size** |
| --- | --- | --- | --- | --- | --- |
| **No-Go < Oddball (blue)** |  | | | | |
| Left Precuneus | 0 | -70 | 53 | 0.03 | 57 |
| Left Postcentral Cortex | -36 | -30 | 53 | 0.01 | 99 |
| **No-Go > Oddball (red)** |  | | | | |
| Right Inferior Frontal Cortex | 40 | 22 | 10 | 0.03 | 46 |
| Right Superior Temporal Cortex | 51 | -59 | 30 | 0.01 | 98 |
| Right Postcentral Cortex | 25 | -30 | 56 | 0.05 | 35 |
| Left Superior Temporal Cortex | -54 | -52 | 7 | 0.02 | 117 |
| Left Medial Frontal Cortex | -11 | 0 | 56 | 0.05 | 38 |

x, y, z = Peak Talairach coordinates. Abbreviations: BOLD, blood-oxygen-level dependent; TD, typically developed controls; ASD, individuals with autism spectrum disorder.

***Supplementary table 8:*** *Anatomical location and statistics for BOLD activation for the Go/No-Go task (ASD group, placebo condition)*

| **Region** | **X** | **Y** | **Z** | **Cluster *p*-value** | **Cluster size** |
| --- | --- | --- | --- | --- | --- |
| **No-Go < Oddball (blue)** |  | | | | |
| Right Cuneus | 11 | -59 | 7 | 0.01 | 113 |
| Right Superior Frontal Cortex | 22 | 41 | 23 | 0.02 | 97 |
| Left Middle Temporal Cortex | -36 | -59 | 7 | 0.03 | 50 |
| Left Medial Frontal Cortex | -11 | 56 | 10 | 0.02 | 94 |
| Left Postcentral Cortex | -36 | -30 | 50 | 0.008 | 197 |
| **No-Go > Oddball (red)** |  | | | | |
| Right Occipital Cortex | 33 | -89 | -17 | 0.02 | 81 |
| Right Precentral Cortex | 47 | -11 | 50 | 0.01 | 129 |
| Right Medial Frontal Cortex | 4 | 7 | 46 | 0.01 | 117 |
| Left Occipital Cortex | -18 | -93 | -17 | 0.01 | 139 |
| Left Middle Temporal Cortex | -58 | -41 | 17 | 0.05 | 38 |
| Left Precuneus | -22 | -78 | 40 | 0.02 | 58 |
| Left Middle Frontal Cortex | -36 | 19 | 46 | 0.02 | 77 |

x, y, z = Peak Talairach coordinates. Abbreviations: BOLD, blood-oxygen-level dependent; TD, typically developed controls; ASD, individuals with autism spectrum disorder.

***Supplementary table 9:*** *Anatomical location and statistics for BOLD activation for the Go/No-Go task (ASD group, citalopram condition)*

| **Region** | **X** | **Y** | **Z** | **Cluster *p*-value** | **Cluster size** |
| --- | --- | --- | --- | --- | --- |
| **No-Go < Oddball (blue)** |  | | | | |
| Right Insula | 36 | -44 | 20 | 0.005 | 248 |
| Right Precuneus | 29 | -67 | 40 | 0.05 | 33 |
| Left Posterior Cingulate Cortex | -25 | -70 | 7 | 0.04 | 38 |
| Left Superior Frontal Cortex | -18 | 63 | 10 | 0.03 | 48 |
| Left Middle Frontal Cortex | -40 | 19 | 26 | 0.008 | 218 |
| Left Inferior Parietal Cortex | -36 | -30 | 40 | 0.002 | 575 |
| **No-Go > Oddball (red)** |  | | | | |
| Right Inferior Frontal Cortex | 51 | 41 | 3 | 0.04 | 26 |
| Right Postcentral Cortex | 51 | -11 | 13 | 0.04 | 21 |
| Right Anterior Cingulate Cortex | 0 | -33 | 23 | 0.03 | 52 |
| Right Superior Frontal Cortex | 7 | 7 | 56 | 0.01 | 101 |

x, y, z = Peak Talairach coordinates. Abbreviations: BOLD, blood-oxygen-level dependent; TD, typically developed controls; ASD, individuals with autism spectrum disorder.

***Supplementary table 10: Anatomical location and statistics for BOLD activation for the sustained attention task (TD group, placebo condition)***

| **Region** | **X** | **Y** | **Z** | **Cluster *p*-value** | **Cluster size** |
| --- | --- | --- | --- | --- | --- |
| **8s > 0.5s (red)** |  |  |  |  |  |
| Right Cuneus | 14 | -93 | 3 | 0.0006 | 5629 |
| Left Middle Temporal Cortes | -54 | -33 | 3 | 0.002 | 572 |
| Left Superior Frontal Cortex | -33 | 48 | 33 | 0.002 | 408 |
| **8s < 0.5s (blue)** |  |  |  |  |  |
| Right Cerebellum | 43 | -74 | -17 | 0.0007 | 6123 |
| Bilateral Medial Frontal Cortex | 0 | -4 | 53 | 0.004 | 441 |

x, y, z = Peak Talairach coordinates. Abbreviations: BOLD, blood-oxygen-level dependent; TD, typically developed controls; ASD, individuals with autism spectrum disorder.

***Supplementary table 11: Anatomical location and statistics for BOLD activation for the sustained attention task (TD group, citalopram condition)***

| **Region** | **X** | **Y** | **Z** | **Cluster *p*-value** | **Cluster size** |
| --- | --- | --- | --- | --- | --- |
| **8s > 0.5s (red)** |  |  |  |  |  |
| Right Superior Temporal Cortex | 51 | -11 | -3 | 0.0007 | 1297 |
| Left Superior Temporal Cortex | -54 | -7 | -7 | 0.005 | 555 |
| Left Lingual Cortex | -4 | -93 | -3 | 0.0007 | 4544 |
| **8s < 0.5 (blue)** |  |  |  |  |  |
| Right Inferior Parietal Cortex | 47 | -59 | 60 | 0.0008 | 578 |
| Left Postcentral Cortex | -43 | -22 | 43 | 0.002 | 344 |
| Left Inferior Occipital Cortex | -40 | -81 | -13 | 0.0008 | 5022 |

x, y, z = Peak Talairach coordinates. Abbreviations: BOLD, blood-oxygen-level dependent; TD, typically developed controls; ASD, individuals with autism spectrum disorder.

***Supplementary table 12: Anatomical location and statistics for BOLD activation for the sustained attention task (ASD group, placebo condition)***

| **Region** | **X** | **Y** | **Z** | **Cluster *p*-value** | | **Cluster size** |
| --- | --- | --- | --- | --- | --- | --- |
| **8s > 0.5s (red)** |  |  |  |  |  | |
| Right Superior Temporal Cortex | 40 | 22 | -30 | 0.004 | 313 | |
| Left Cerebellum | -7 | -78 | -10 | 0.0006 | 4256 | |
| Left Middle Temporal Cortex | -58 | -41 | 0 | 0.006 | 237 | |
| **8s < 0.5 (blue)** |  |  |  |  |  | |
| Right Inferior Frontal Cortex | 58 | 19 | 23 | 0.002 | 412 | |
| Left Postcentral Cortex | -36 | -30 | 56 | 0.0006 | 1148 | |
| Left Cerebellum | -33 | -89 | -20 | 0.0006 | 2656 | |

x, y, z = Peak Talairach coordinates. Abbreviations: BOLD, blood-oxygen-level dependent; TD, typically developed controls; ASD, individuals with autism spectrum disorder.

***Supplementary table 13: Anatomical location and statistics for BOLD activation for the sustained attention task (ASD group, citalopram condition)***

| **Region** | **X** | **Y** | **Z** | **Cluster *p*-value** | **Cluster size** |
| --- | --- | --- | --- | --- | --- |
| **8s > 0.5s (red)** |  |  |  |  |  |
| Right Postcentral Cortex | 58 | -11 | 23 | 0.004 | 187 |
| Right Lingual Cortex | 11 | -93 | -7 | 0.0005 | 3525 |
| **8s < 0.5 (blue)** |  |  |  |  |  |
| Right Fusiform Cortex | 47 | -63 | -10 | 0.0005 | 435 |
| Right Precuneus | 29 | -74 | 30 | 0.0005 | 2000 |
| Left Cerebellum | -36 | -85 | -20 | 0.0005 | 471 |
| Left Precentral Cortex | -33 | -30 | 56 | 0.0005 | 1081 |

x, y, z = Peak Talairach coordinates. Abbreviations: BOLD, blood-oxygen-level dependent; TD, typically developed controls; ASD, individuals with autism spectrum disorder.

## Supplementary figure 1


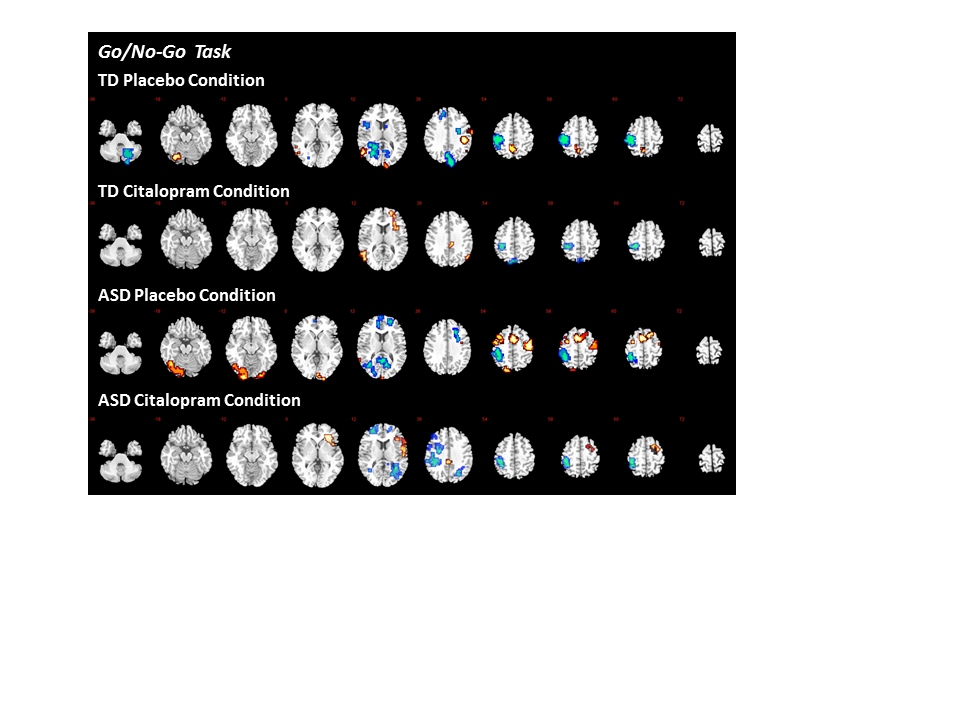


Anatomical location and statistics for BOLD activation per group and drug condition for Go/No-Go task. Locations of group-wise BOLD signals from No-Go versus Oddball and 0.5s versus 8s contrasts. Go/No-Go task: Red = No-Go > oddball. Blue = No-Go < oddball. For Sustained Attention Task: Red = 8s > 0.5s. Blue = 0.5s > 8s. Numeric label = z Talairach coordinate. Right hemisphere of brain is on the right side of the image. Abbreviations: BOLD, blood-oxygen-level dependent; TD, Typically Developed Controls; ASD, Individuals with Autism Spectrum Disorder.

## Supplementary figure 2


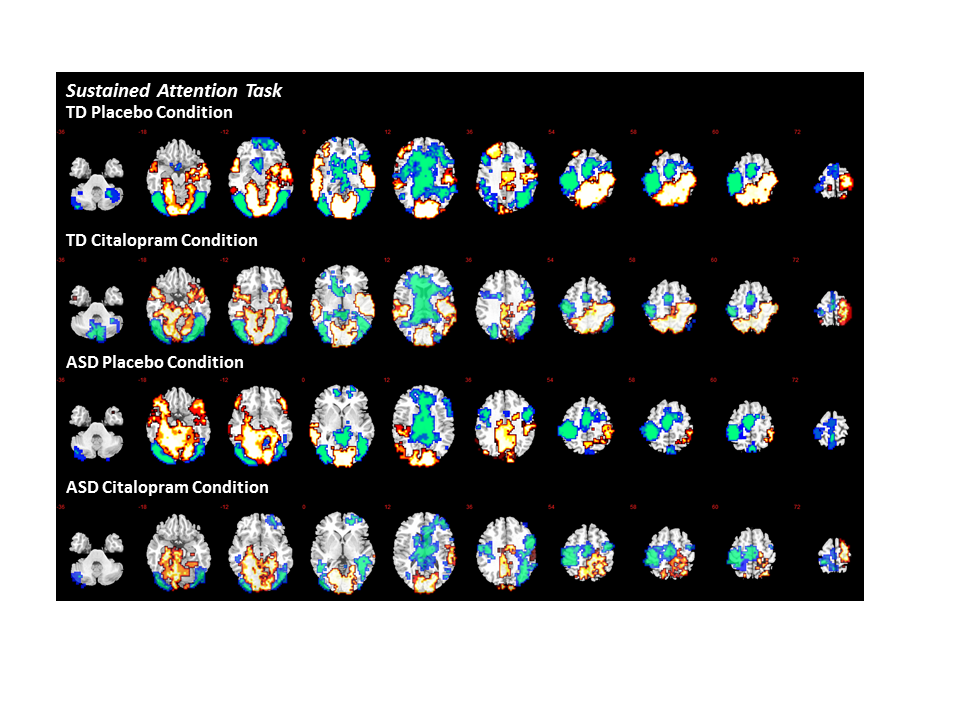


Anatomical location and statistics for BOLD activation per group and drug condition for Sustained Attention task. Locations of group-wise BOLD signals from No-Go versus Oddball and 0.5s versus 8s contrasts. Go/No-Go task: Red = No-Go > oddball. Blue = No-Go < oddball. For Sustained Attention Task: Red = 8s > 0.5s. Blue = 0.5s > 8s. Numeric label = z Talairach coordinate. Right hemisphere of brain is on the right side of the image. Abbreviations: BOLD, blood-oxygen-level dependent; TD, Typically Developed Controls; ASD, Individuals with Autism Spectrum Disorder.

## Supplementary figure 3


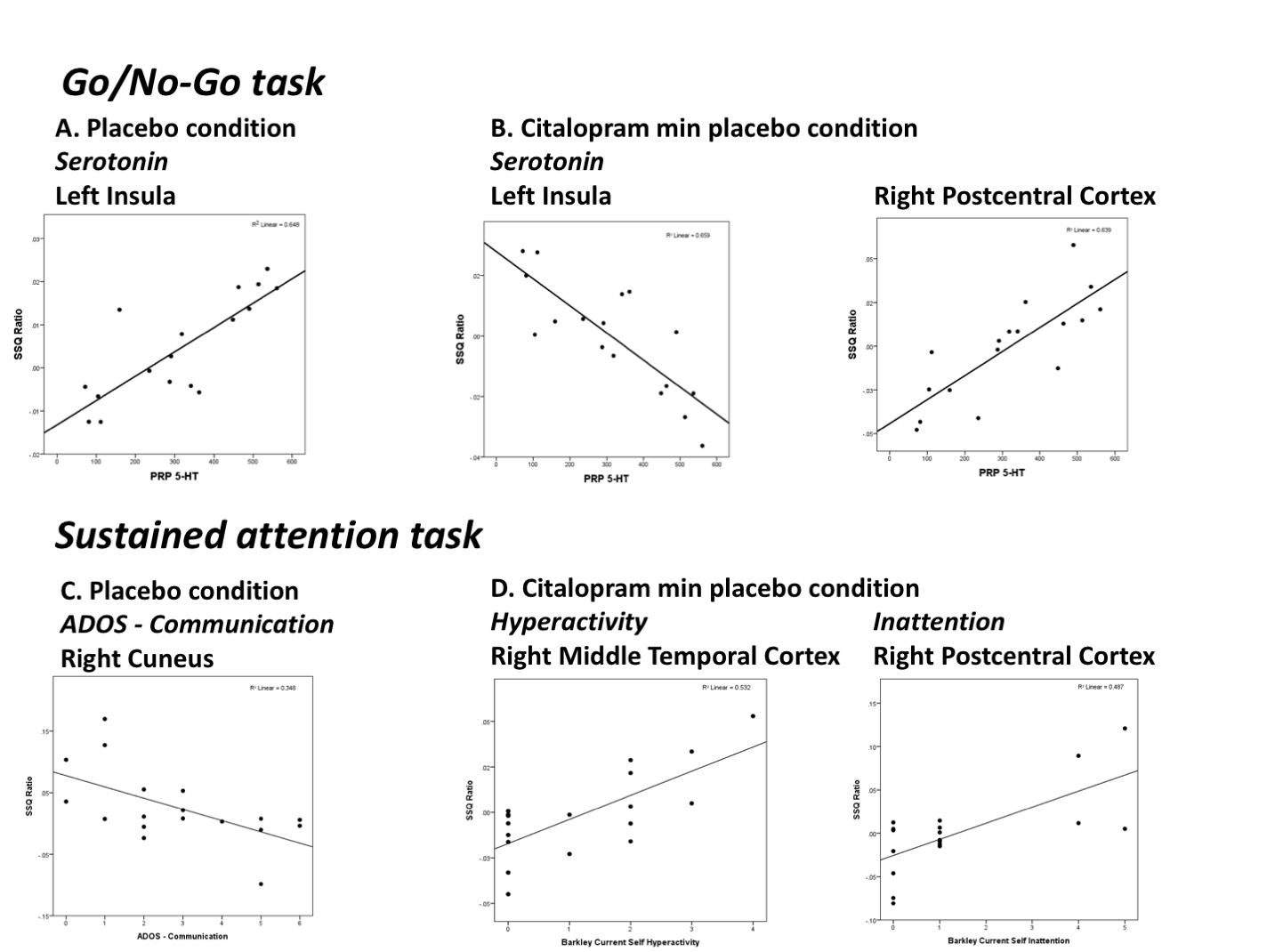


Correlations of No-Go versus Oddball contrast BOLD response (citalopram – placebo) and PRP 5-HT levels and correlations of 0.5s versus 8s contrast BOLD response (citalopram – placebo) and ADHD symptoms (n=18). Right hemisphere of brain is on the right side of the image. Abbreviations: SSQ, sum of squares (statistical measure of BOLD response); PRP 5-HT, platelet rich plasma serotonin (ug/l); BOLD, blood-oxygen-level dependent.

# References

1 Brammer, M. J. *et al.* Generic brain activation mapping in functional magnetic resonance imaging: a nonparametric approach. *Magnetic resonance imaging* **15**, 763-770 (1997).

2 Bullmore, E. T. *et al.* Methods for diagnosis and treatment of stimulus-correlated motion in generic brain activation studies using fMRI. *Hum. Brain Mapp.* **7**, 38-48 (1999).

3 Friman, O., Borga, M., Lundberg, P. & Knutsson, H. Adaptive analysis of fMRI data. *Neuroimage* **19**, 837-845 (2003).

4 Bullmore, E. *et al.* Colored noise and computational inference in neurophysiological (fMRI) time series analysis: Resampling methods in time and wavelet domains. *Hum. Brain Mapp.* **12**, 61-78 (2001).

5 Bullmore, E. T. *et al.* Global, voxel, and cluster tests, by theory and permutation, for a difference between two groups of structural MR images of the brain. *Medical Imaging, IEEE Transactions on* **18**, 32-42 (1999).

6 Talairach, J. & Tournoux, P. *Co-Planar Stereotaxic Atlas of the Human Brain: Three- Dimensional Proportional Systems*. (Thieme Medical 1988).

7 Brammer, M. J. *et al.* Generic brain activation mapping in functional magnetic resonance imaging: A nonparametric approach. *Magn. Reson. Imaging* **15**, 763-770 (1997).
